# Near-fatal asthma in a 12-year-old girl leading to life-threatening tonsillar herniation: a case report

**DOI:** 10.1186/s13256-025-05507-5

**Published:** 2025-09-01

**Authors:** Abdullah Almutairi, Khalid Althobaiti, Mohannad Antar, Hala Al Alem, Amna Kashgari

**Affiliations:** 1 Department of Pediatrics, King Abdullah Specialist Children’s Hospital (KASCH), National Guard Health Affairs (NGHA), Riyadh, Saudi Arabia; 2 King Abdullah International Medical Research Center (KAIMRC), Ministry of National Guard, Riyadh, Saudi Arabia; 3 College of Medicine, King Saud Bin Abdulaziz University for Health Sciences (KSAU-HS), Riyadh, Saudi Arabia; 4 Department of Radiology, King Abdullah Specialist Children’s Hospital (KASCH), National Guard Health Affairs (NGHA), Riyadh, Saudi Arabia

**Keywords:** Status asthmaticus, Tonsillar herniation, Cerebral edema, Neurological complications, Pediatric critical care

## Abstract

**Background:**

Severe asthma exacerbations can lead to rare and life-threatening complications such as cerebral edema and tonsillar herniation. This case highlights the importance of early recognition, aggressive treatment, and the implementation of standardized pediatric intensive care unit protocols for managing critical asthma complications.

**Case presentation:**

We report the case of a 12-year-old girl of Middle Eastern descent from Saudi Arabia with a history of bronchial asthma and allergic rhinitis who developed cerebral edema and resultant tonsillar herniation following a severe asthma exacerbation. Her presentation was marked by respiratory distress unresponsive to initial therapy. Intensive management in the pediatric intensive care unit including mechanical ventilation and neuroprotective measures resulted in full neurological recovery prior to discharge.

**Conclusion:**

This case underscores the need for prompt identification and multidisciplinary management of severe asthma complications in pediatric patients to prevent irreversible outcomes.

## Introduction

Asthma is a chronic, heterogeneous inflammatory disease characterized by recurrent symptoms such as wheezing, shortness of breath, chest tightness, and coughing. These symptoms vary over time and in intensity, often accompanied by intermittent airflow obstruction [1, 2]. Status asthmaticus, a severe and potentially fatal exacerbation of asthma, is marked by persistent bronchial obstruction unresponsive to standard treatments, leading to respiratory failure. Near-fatal asthma exacerbations can result in hypercapnia, hypoxemia, and respiratory arrest, primarily due to asphyxia rather than cardiac arrhythmias [3, 4].

Asthma is the most common chronic disease in children, with a prevalence of 10.3–13.3% in Saudi Arabia over the past two decades [5, 6]. Asthma exacerbations are a major cause of hospitalizations in children, and severe cases requiring pediatric intensive care unit (PICU) admission are increasing globally [7, 8]. Although guidelines such as the Global Initiative for Asthma (GINA) exist for asthma management, they provide limited guidance for pediatric intensive care unit (PICU)-specific scenarios [9, 10]. The lack of standardized protocols in the PICU setting can lead to delayed treatment adjustments and prolonged ICU stays [9–11] Standard treatments for severe asthma exacerbations include continuous nebulized beta-agonists, systemic corticosteroids, and intravenous magnesium sulfate [10–13]. However, the efficacy of alternative therapies such as noninvasive positive pressure ventilation and helium–oxygen mixtures remains unclear [10, 14, 15]. Clinical respiratory scoring has shown promise in standardizing assessments and improving coordination among healthcare providers [16, 17]. Despite these advances, cases of refractory asthma requiring invasive interventions such as intubation remain challenging due to associated complications. Although extremely rare, cerebral complications such as tonsillar herniation can occur in status asthmaticus due to profound hypoxia and hypercapnia [18–20]. We describe a case of near-fatal asthma complicated by tonsillar herniation, an atypical complication.

## Case presentation

A 12-year-old girl of Middle Eastern descent from Saudi Arabia, with a history of bronchial asthma and allergic rhinitis, reportedly poorly controlled due to inconsistent use of inhaled corticosteroids and lack of recent follow-up, presented with high-grade fever and shortness of breath. She lived with both parents in a supportive home environment and had no known psychosocial stressors. There was no family history of asthma, neurological disorders, or other chronic illnesses. Her symptoms began after exposure to her mother, who had an upper respiratory tract infection. In the emergency department, she showed signs of severe respiratory distress, with a respiratory rate of 30 breaths per minute, heart rate of 164 beats per minute, oxygen saturation of 95% on room air, and blood pressure of 119/65 mmHg. Initial treatment included nebulized salbutamol, intravenous dexamethasone, and ceftriaxone. However, her respiratory condition continued to deteriorate, necessitating transfer to a higher-level care facility.

Upon arrival at the tertiary center, she was stable on 2 L of oxygen via nasal cannula. However, her condition worsened over the next few hours, with her oxygen saturation suddenly dropping to 75%. She was switched to a non-rebreather mask at 15 L per minute, but her oxygenation remained critically low, prompting resuscitation efforts.

Diagnosed with a severe asthma exacerbation, she was treated with intravenous methylprednisolone, intramuscular epinephrine, continuous nebulized salbutamol, magnesium sulfate, and an intravenous fluid bolus. Despite these interventions, her respiratory distress worsened significantly, leading to her admission to the PICU, where she required high-flow nasal cannula (HFNC) support, continuous salbutamol nebulizer, and intravenous salbutamol. As her respiratory failure progressed and her consciousness declined, she was intubated and sedated with ketamine, fentanyl, and cisatracurium.

**Fig. 1:**
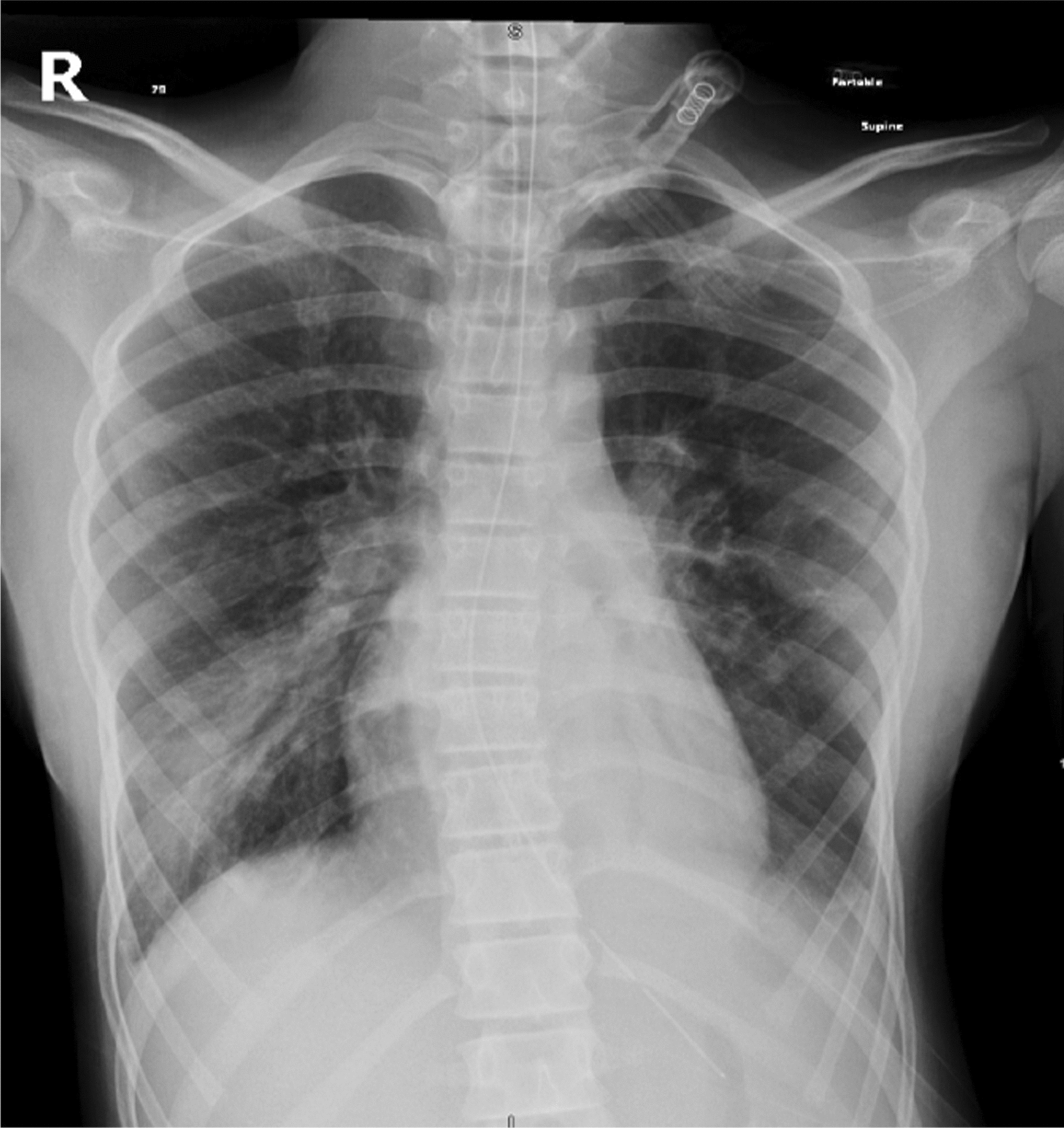
Chest x-ray showing bilateral hyperinflation of the lung and right middle lobe infiltration

Chest x-ray after intubation (Fig. 1) showed bilateral hyperinflation of the lung and right middle lobe infiltration.

**Fig. 2:**
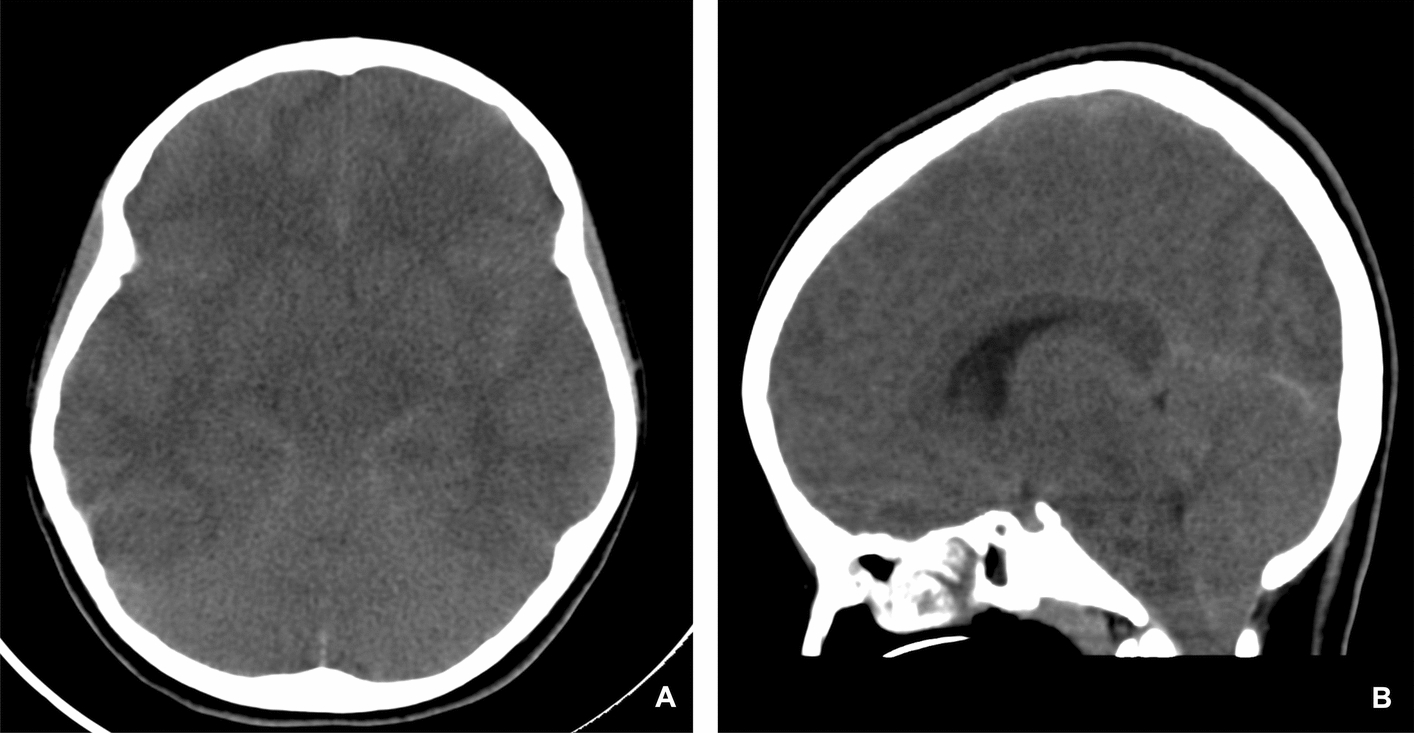
**A** Axial unenhanced computed tomography brain showing diffuse brain edema with effacement of the sulci and subarachnoid spaces. **B** Sagittal unenhanced computed tomography brain showing tonsillar herniation

**Fig. 3:**
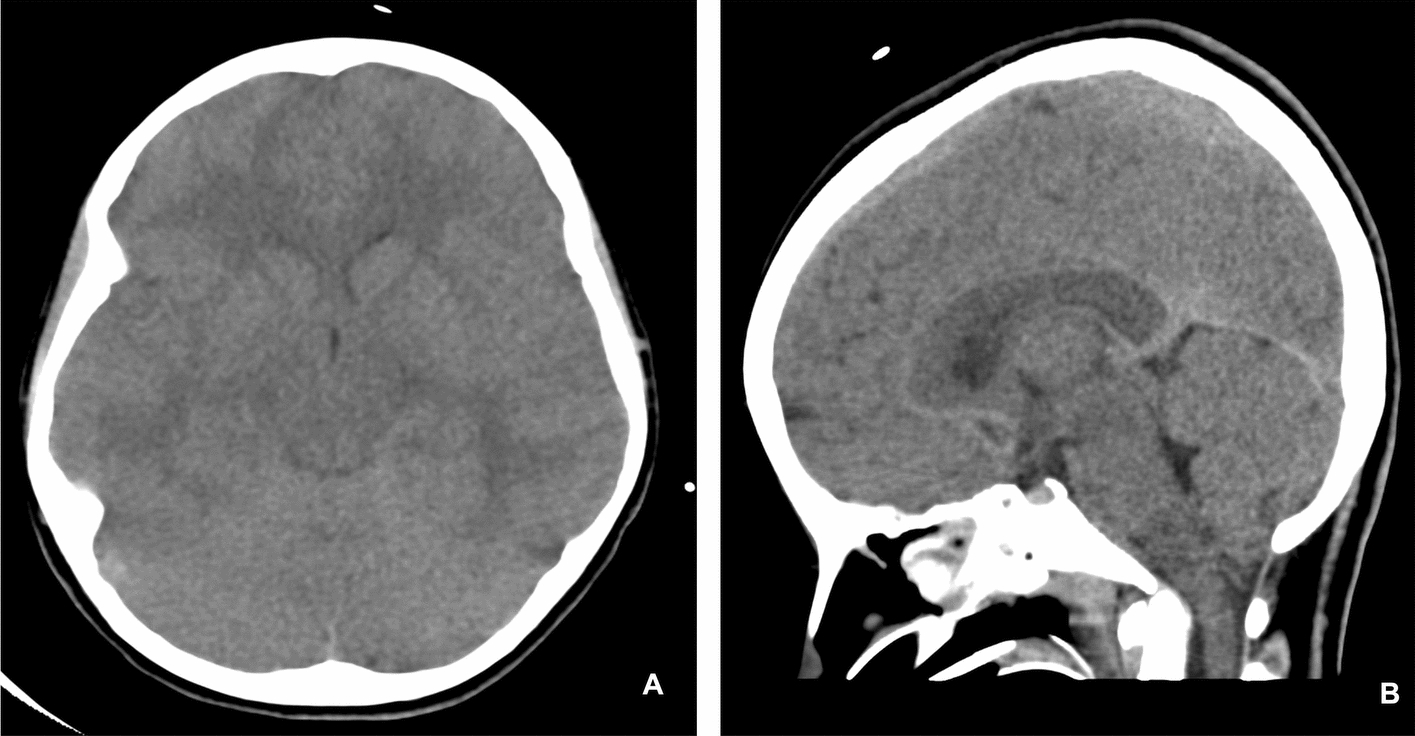
Unenhanced computed tomography scan follow-up in 24 hours **A** Axial image showing interval improvement of the brain edema. **B** Sagittal image showing interval improvement of the tonsillar herniation

While on mechanical ventilation, the patient experienced persistently high peak pressures (up to 60 cm H_2_O) and plateau pressures of 30 cm H_2_O, with significant auto-positive end-expiratory pressure (PEEP) of 25 cm H_2_O. Arterial blood gas analysis showed severe respiratory acidosis, with PCO_2_ levels ranging from 89 mmHg to 117 mmHg and a pH consistently below 7.05, despite frequent ventilator adjustments. During her PICU stay, she tested positive for influenza A via nasopharyngeal aspirate (NPA) and coronavirus disease 2019 (COVID-19) via rapid antigen test. Oseltamivir was initiated due to a positive nasopharyngeal polymerase chain reaction (PCR) for influenza A, which was suspected as a potential trigger for the asthma exacerbation. Despite this, her neurological condition deteriorated within 24 hours, with her Glasgow Coma scale (GCS) dropping to 3/15 and the appearance of fixed, dilated pupils. Aggressive cerebral edema management was initiated, including boluses of 3% NaCl, mannitol, and manual hyperventilation. Computed tomography (CT) scan (Fig. 2) revealed diffuse cerebral and cerebellar edema with tonsillar herniation. On the basis of the clinical context at the time, the herniation was presumed to be secondary to prolonged hypercapnia, hypoxia, and impaired cerebral venous return, resulting in elevated intracranial pressure (ICP). After sedation was discontinued, her GCS improved slightly to 7/15, with some spontaneous movements and a sluggish reaction in the left pupil (2 mm), while the right pupil remained fixed at 4 mm. Neurosurgical consultation ruled out the need for surgical intervention, and neuroprotective measures, including controlled PCO_2_, serum sodium levels between 145 mmol/L and 150 mmol/L, mean arterial pressure (MAP) above 65 mmHg, and deep sedation, were maintained. Echocardiogram revealed a patent foramen ovale (PFO) with a left-to-right shunt but no other significant findings. Repeat brain CT (Fig. 3) after 24 hours showed improvement in cerebral and cerebellar edema, with the tonsillar herniation reduced from 10 mm to 5.5 mm at the foramen magnum.

**Fig. 4:**
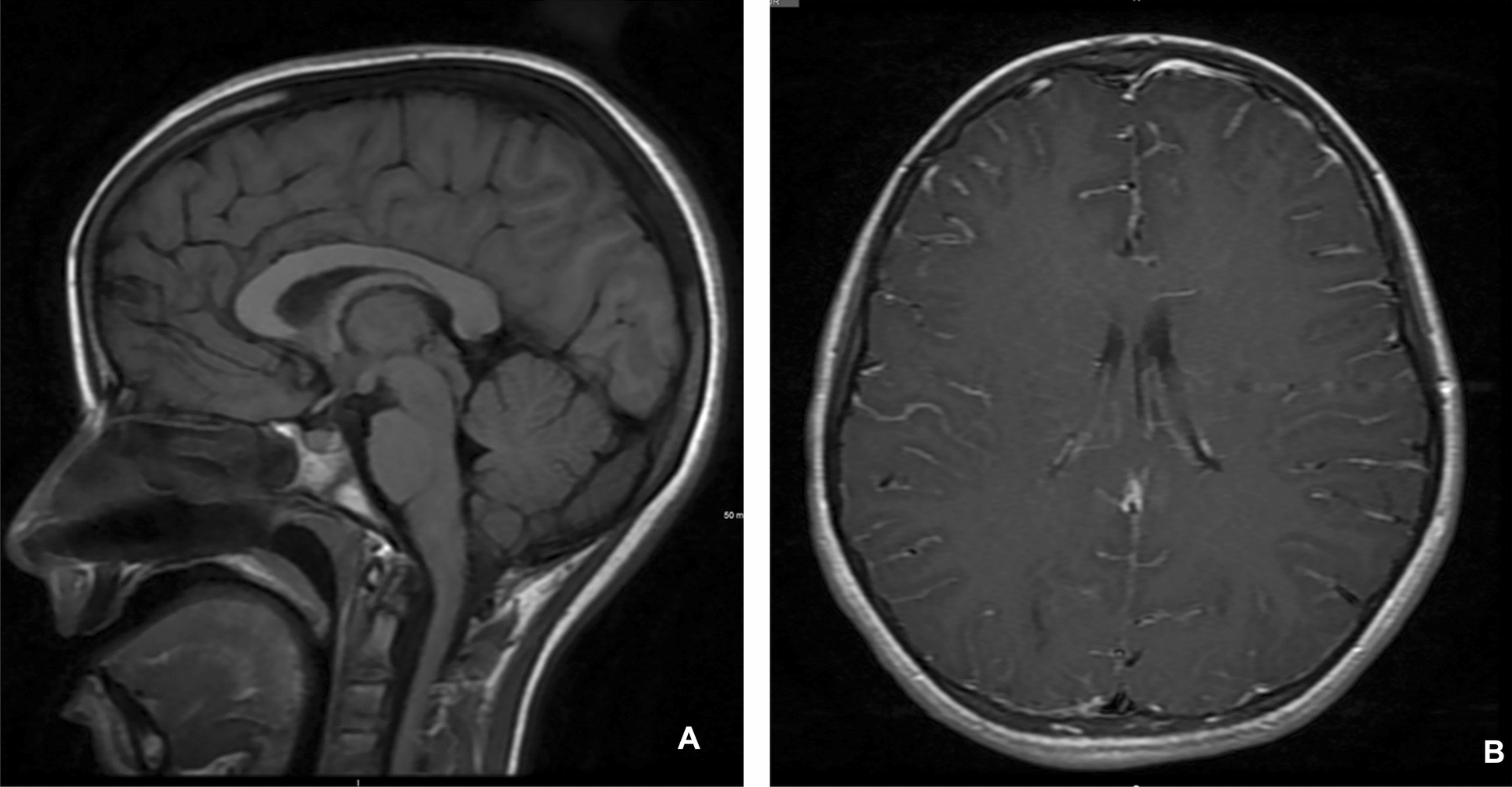
**A** Unenhanced sagittal T1WI showing no evidence of tonsillar herniation. **B** Enhanced axial T1WI showing diffuse gyriform or serpentine enhancement in keeping with leptomeningeal enhancement

**Fig. 5:**
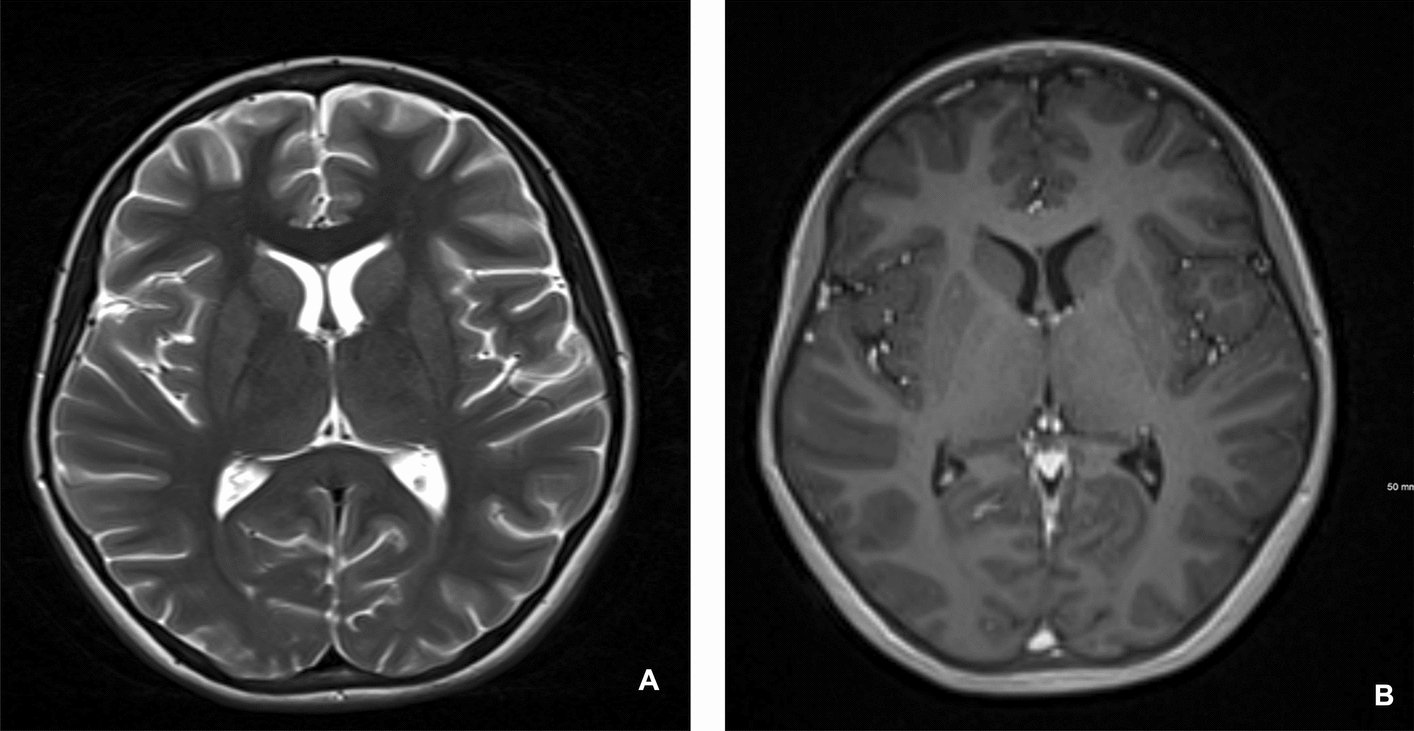
**A** Unenhanced axial T2WI showing normal brain parenchyma with no residual brain edema. **B** Enhanced axial T1WI showing complete resolution of the leptomeningeal enhancement

Further, 2 days later, magnetic resonance imaging (MRI) (Fig. 4) revealed cortical and subcortical abnormalities, suggestive of septic emboli, along with diffuse leptomeningeal enhancement, indicating an infectious process. Cerebral edema and tonsillar herniation had resolved, and magnetic resonance angiography (MRA) and venography (MRV) were normal. Neuroprotective measures were discontinued following the MRI findings. In the days that followed, the patient’s respiratory status improved, allowing salbutamol nebulization to be spaced to every 3 hours. She was successfully extubated after 6 days and transitioned to nasal cannula oxygen. She completed a 10-day course of ceftriaxone and oseltamivir, along with a 1-week course of methylprednisolone. The patient developed right eye ptosis following extubation. Third nerve palsy was ruled out after ophthalmologic evaluation. The ptosis resolved completely within 1 week, and no long-term neurological deficits were observed. After 13 days the patient was discharged home with follow-up brain MRI (Fig. 5). She was seen in the clinic 1 week later and had returned to her baseline status prior to the illness. At 3-month follow-up with both pulmonology and neurology clinics, the patient’s asthma was well controlled with appropriate medications, and she had returned to her neurological baseline with no residual deficits. A summary of the patient’s vital signs, major interventions, and clinical progression is provided in Table 1.

**Table 1:** Summary of patient’s vital signs, major interventions, and clinical progression

Timepoint	Vitals and labs	Interventions	Comments
Emergency department (ED) arrival (day 0)	RR: 30 breaths per minute, HR: 164 beats per minute, BP: 119/65 mmHg, SpO_2_: 95% room air, GCS: 15/15	Nebulized salbutamol, intravenous dexamethasone, ceftriaxone	Initial management at referring hospital
Tertiary transfer (day 1)	Stable on 2 L nasal cannula	Intravenous methylprednisolone, magnesium sulfate, intramuscular epinephrine	SpO_2_ dropped to 75% after 6 hours
PICU admission (day 1)	SpO_2_: 75% room air, GCS: 15/15, pH: 7.33, pCO_2_: 35, HCO_3_: 18	HFNC, continuous nebulized salbutamol, intravenous salbutamol	Respiratory distress and consciousness deteriorated after 4 hours
Intubation and sedation (day 1)	PIP: 60, Pplat: 30, auto-PEEP: 25, pH: 6.93, pCO_2_: 117, HCO_3_: 24	Ketamine, fentanyl, cisatracurium, oseltamivir	Severe respiratory failure, COVID-19, and influenza A positive
Neuro deterioration (day 2)	GCS: 3/15, fixed dilated pupils, pH: 7.27, pCO_2_: 59, HCO_3_: 27	3% NaCl, mannitol, manual hyperventilation	Brain CT: tonsillar herniation
Improvement in imaging (day 3)	Vitally stable with normal blood gas; GCS 7/15	Continued neuroprotective measures	Brain CT: improved cerebral edema, reduced herniation
Extubation and recovery (days 4–6)	GCS 14/15, ptosis after extubation	Asthma well controlled, neurologically at baseline	MRI: resolution of edema and herniation
Transfer to the ward and discharge (days 7–13)	Vitally stable weaned to room air, ptosis resolved	Spaced salbutamol, complete ceftriaxone and oseltamivir	No neurological sequelae
Follow-up (1 week and 3 months)	Stable, neurologically intact, asthma controlled	Inhaled corticosteroids, routine pulmonology and neurology follow-up	Returned to baseline; no residual deficits

## Discussion

This case highlights the challenges of managing severe asthma exacerbations complicated by cerebral edema and resultant tonsillar herniation. The patient, a 12-year-old girl of Middle Eastern descent with poor compliance to asthma medications, deteriorated rapidly despite receiving standard asthma management. Her presentation underscores the importance of early recognition, aggressive therapy, and multidisciplinary approach in cases of status asthmaticus [3, 5, 8].

The management of this patient included standard interventions such as continuous nebulized beta-agonists, systemic corticosteroids, and magnesium sulfate. These treatments align with established guidelines and are supported by robust evidence [10, 12, 13]. HFNC was initiated to manage hypoxemia, consistent with recommendations for noninvasive support in severe asthma [14]. However, the patient’s worsening condition necessitated intubation and mechanical ventilation. Permissive hypercapnia was applied post-intubation, which has been shown to reduce mortality in severe asthma cases by minimizing barotrauma and hyperinflation [21–23].

Although cerebral edema is an extremely rare complication of asthma, several case reports have documented its occurrence, likely secondary to profound hypercapnia and hypoxia. Masuda *et al*. described life-threatening hypercapnia leading to cerebral complications in an infant with status asthmaticus managed with isoflurane [18]. Similarly, Rodrigo *et al*. reported a case of subarachnoid hemorrhage following permissive hypercapnia in a patient with severe acute asthma, underscoring the potential risks associated with aggressive ventilatory strategies [19]. These reports support the hypothesis that sustained elevations in carbon dioxide and impaired cerebral venous return can predispose to elevated intracranial pressure and cerebral herniation, as observed in our patient. Viral infections, including influenza A and COVID-19, likely exacerbated the inflammatory response, as evidenced by leptomeningeal enhancement on imaging [24]. Aggressive management of cerebral edema with hypertonic saline and mannitol stabilized her condition, allowing recovery without permanent neurological deficits.

This case underscores the need for standardized PICU asthma management protocols to address severe complications. Implementing critical care asthma pathways (CCAPs) could reduce variability in care and improve outcomes [11, 16, 17]. Furthermore, attention for neurological complications in patients with refractory asthma is essential, as prompt recognition and treatment can prevent catastrophic outcomes [18].

## Conclusion

Early recognition of life-threatening asthma exacerbations and timely, aggressive management are critical in preventing catastrophic outcomes such as brain edema and herniation. Rapid escalation of therapy, close monitoring, and preparedness for potential complications can significantly improve patient outcomes. This case underscores the importance of attentive care in severe asthma cases to mitigate the risk of irreversible damage.

## Data Availability

Not applicable as no datasets were generated or analyzed.

## Abbreviations

PICU: Pediatric intensive care unit
GINA: Global Initiative for Asthma
IV: Intravenous
IM: Intramuscular
PEEP: Positive end-expiratory pressure
NPA: Nasopharyngeal aspirate
COVID-19: Coronavirus disease 2019
GCS: Glasgow Coma scale
CT: Computed tomography
PCO_2_: Partial pressure of carbon dioxide
MAP: Mean arterial pressure
ICP: Intracranial pressure
PFO: Patent foramen ovale
MRI: Magnetic resonance imaging
MRA: Magnetic resonance angiography
MRV: Magnetic resonance venography
HFNC: High-flow nasal cannula
CCAPs: Critical care asthma pathways
